# Simple PCR Assays Improve the Sensitivity of HIV-1 Subtype B Drug Resistance Testing and Allow Linking of Resistance Mutations

**DOI:** 10.1371/journal.pone.0000638

**Published:** 2007-07-25

**Authors:** Jeffrey A. Johnson, Jin-Fen Li, Xierong Wei, Jonathan Lipscomb, Diane Bennett, Ashley Brant, Mian-er Cong, Thomas Spira, Robert W. Shafer, Walid Heneine

**Affiliations:** 1 Laboratory Branch, Division of HIV/AIDS Prevention, National Center for HIV/AIDS, Viral Hepatitis, STD, and TB Prevention, Centers for Disease Control and Prevention, Atlanta, Georgia, United States of America; 2 HIV Incidence and Case Surveillance Branch, National Center for HIV/AIDS, Viral Hepatitis, STD, and TB Prevention, Centers for Disease Control and Prevention, Atlanta, Georgia, United States of America; 3 Global AIDS Program, National Center for HIV/AIDS, Viral Hepatitis, STD, and TB Prevention, Centers for Disease Control and Prevention, Atlanta, Georgia, United States of America; 4 Stanford University Medical Center, Stanford, California, United States of America; HIV/AIDS Clinical Research Unit, Brazil

## Abstract

**Background:**

The success of antiretroviral therapy is known to be compromised by drug-resistant HIV-1 at frequencies detectable by conventional bulk sequencing. Currently, there is a need to assess the clinical consequences of low-frequency drug resistant variants occurring below the detection limit of conventional genotyping. Sensitive detection of drug-resistant subpopulations, however, requires simple and practical methods for routine testing.

**Methodology:**

We developed highly-sensitive and simple real-time PCR assays for nine key drug resistance mutations and show that these tests overcome substantial sequence heterogeneity in HIV-1 clinical specimens. We specifically used early wildtype virus samples from the pre-antiretroviral drug era to measure background reactivity and were able to define highly-specific screening cut-offs that are up to 67-fold more sensitive than conventional genotyping. We also demonstrate that sequencing the mutation-specific PCR products provided a direct and novel strategy to further detect and link associated resistance mutations, allowing easy identification of multi-drug-resistant variants. Resistance mutation associations revealed in mutation-specific amplicon sequences were verified by clonal sequencing.

**Significance:**

Combined, sensitive real-time PCR testing and mutation-specific amplicon sequencing provides a powerful and simple approach that allows for improved detection and evaluation of HIV-1 drug resistance mutations.

## Introduction

Highly active antiretroviral therapy (HAART) can provide sustained clinical benefit for HIV-1 infected persons, but treatment success is jeopardized by drug resistance. Drug resistance testing supports the management of persons on HAART and is recommended to help guide treatment choices [1]. Resistance-related mutations, however, are conventionally detected by bulk sequence analysis of viral RNA from plasma which does not reliably detect variants comprising less than 20% of the viruses in a sample [2]. Identifying drug-resistant variants at frequencies below the detection capability of conventional genotyping requires new diagnostic methods. Currently, there is increasing recognition that identification of low-frequency drug-resistant viruses is vital for evaluating the full clinical impact of drug resistance and for understanding the dynamics of drug resistance emergence and persistence [3]–[7].

A few seminal studies illustrated the advantages of sensitive drug resistance assays with women who received intrapartum single-dose nevirapine (SD-NVP). These reports on sensitive testing for nevirapine resistance have shown that drug resistance emerges more frequently and persists longer than previously demonstrated by conventional sequencing [3], [4], [5]. Persisting minority nevirapine-resistant viruses may contribute to poor virologic responses when subsequent regimens contain nevirapine-related drugs [6], [7]. Accurate accounting of transmitted drug resistance is also a concern. Because of reduced fitness in the absence of antiretroviral treatment, transmitted drug-resistant variants can decay to levels that are undetectable by conventional sequence analysis [8], [9], [10]. Therefore, the ability to detect low-frequency variants would allow for more informed decisions on the selection of active drugs for both drug-naïve and drug-experienced persons beginning new treatment regimens.

Early hybridization methods to improve HIV-1 resistance mutation detection, such as the Line Probe Assay (LiPA), offered a modest improvement in sensitivity over bulk sequencing but experienced frequent detection failures due to the considerable nucleotide polymorphisms present in HIV-1 [11]. More recent point-mutation assays offer substantial improvements in sensitivity [12], [13], [14] and, previously, we had shown that real-time PCR assays can be both highly specific and sensitive with subtype C viruses from SD-NVP-experienced women [3]. However, point-mutation assays are susceptible to polymorphisms and their performance with large-scale clinical testing is unclear. Moreover, point-mutation testing is inherently limited in its genotypic information because it does not detect mutations beyond what is interrogated by the assay. Other approaches that evaluate numerous virus sequences per sample are highly-informative research tools but are too complex or costly for routine clinical testing [15], [16], [17].

To both simplify and improve the sensitivity of HIV drug resistance testing, we describe a strategy that combines real-time PCR point-mutation assays and direct sequencing of resistance mutation-specific PCR products to identify and link additional mutations. For this purpose, we focused on developing and validating nine assays for key drug resistance mutations in subtype B HIV-1 clinical specimens as a basis for later expansion to other virus mutations and subtypes. Because these assays can detect nearly 2-logs less mutant virus than conventional bulk sequencing they are able to detect resistance-associated mutations that might emerge at low frequencies as part of the normal viral quasispecies [18]. Therefore, to maximize specificity, we used pre-antiretroviral (pre-ARV) drug era wildtype virus samples to define cutoffs that exclude the detection of naturally-occurring minority mutants. We also used primer mixtures and designed mismatches to circumvent the genomic plasticity of HIV-1 and show high sensitivity on a large panel of clinical samples. We demonstrate that these assays are able to detect low-frequency drug resistance and permit easy identification of linked mutations, thus, providing a practical strategy for routine drug resistance testing.

## Methods

### Virus template amplification

HIV-1 genomic RNA was extracted (Qiagen UltraSens RNA kit) from 200 µL plasma or serum and reconstituted in 50 µL of buffer provided with the kit. To ensure sufficient template for repeat testing, virus sequences were first amplified from 5 µL HIV-1 RNA by reverse transcriptase-polymerase chain reaction (RT-PCR) using the reverse primer RTP-REV2 [5′-CTT CTG TAT GTC ATT GAC AGT CC], and forward primer RTP-F1 [5′-CCT CAG ATC ACT CTT TGG CAA CG], which span from n.t. 1 in protease to n.t. 777 in RT. PCR amplification conditions were 40 cycles of 95°C for 45 seconds, 50°C for 30 seconds, and 72°C for 2 minutes. To better evaluate the success of amplifying each sample, the reverse transcriptase and PCR amplification steps were performed separately. We later assessed the validated procedures could be combined into a one-step RT-PCR to reduce specimen handling (not shown). When only RT template was desired, a shorter amplicon generated by the primer pair, RTP-REV2 and RTP-F2 [5′-AAA GTT AAA CAA TGG CCA TTG ACA G] (n.t. 58 to 777 in RT), was used and occasionally provided improved amplification sensitivity. Both primer sets were also successful in generating amplified virus template from proviral sequences (not shown).

### Real-time PCR

**Figure 1:**
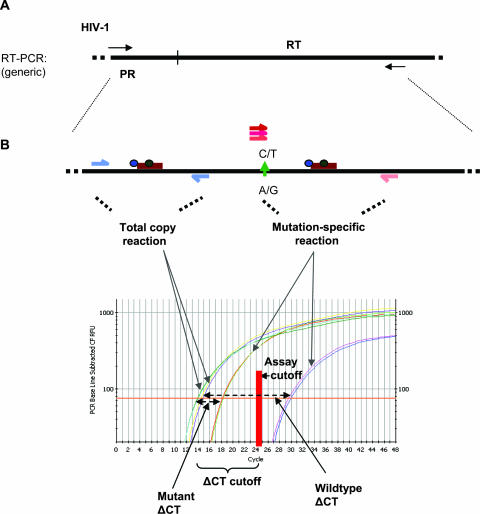
Principle of the real-time PCR assay. A. HIV-1 template generated from RT-PCR of viral RNA is subjected to both total copy and mutation-specific real-time reactions. B. The difference in the total copy and mutation-specific reactions (ΔCT) is used to differentiate mutant and wildtype specimens. In this example, the experimental cutoff is a ΔCT of 10.5 cycles. A mutation-specific CT within 10.5 cycles of the total copy reaction CT would indicate the presence of mutant virus.

Real-time PCR-based mutation-specific assays were developed for the protease L90M and the reverse transcriptase M41L, K65R, K70R, K103N, Y181C, M184V, and both T215Y and F resistance-associated mutations in HIV-1 subtype B. Mutation testing was performed in 96-well plates using 2 µL of 1∶20 dilutions of the RT-PCR products, except that samples with viral loads below 5000 copies/mL were not diluted. The principle of the real-time PCR assay is to compare the differential amplifications of a mutation-specific PCR and a PCR that amplifies all viruses in the sample (total virus copy reaction) (Figure 1). The HIV-1 total copy primers, ComFWD and ComREV, span n.t. 258–420 in RT and were used with the common probes, com1P and 2P (Figure 1A, see Table 1). The same common reaction was used for all resistance mutation tests to reduce labor and costs. For multiple mutation screening, several resistance mutation-specific reactions can be performed simultaneously. The cycle number at which the fluorescence emission exceeds the background fluorescence threshold is the threshold cycle (CT) and is the unit of measure for comparing the differences in amplification signals (ΔCT) between the total copy and mutation-specific reactions (Figure 1B). All samples were tested in duplicate with the means of the total copy and mutation-specific CTs used for the determination of the ΔCT.

**Table 1:** Oligonucleotides for real-time PCR drug resistance testing.

	Oligonucleotide sequence	Proportion
Total copy reaction	ComFWD 5′-CTT CTG GGA AGT TCA ATT AGG AAT ACC	
	ComREV 5′-TGG TGT CTC ATT GTT TRT ACT AGG TA	
	Com 1P 5′-*FAM*-TGG ATG TGG GTG A“T”G CAT ATT TYT CAR TTC CCT TA	60%
	Com 2P 5′-*FAM*-TAC TGG ATG “T” GGG TGA TGC ATA TTT TTC ART TCC CTT A	40%
Mutation		
*Protease*		
L90M	Rev1 a 5′-GAA AAT TTA AAG TGC AAC CAA KTT GAG TGA T	-
	Fwd 5′-AGA TCA CTC TTT GGC AAC GAC C	-
	P1 5′-*FAM*-TAG GGG GAA “T”TG GAG GTT TTR TCA AAG TAA GAC AGT AT	-
*Reverse transcriptase*		
M41L	F1 5′-AAT AAA AGC ATT ART RGA AAT YTG TRC AGC AT	35%
	F2 5′-AAT WAA AGC ATT ART RGA AAT YTG TRC WGC AT	10%
	F3 5′-AAA AGC ATT ART RGA AAT YTG TRC AGG AC	32%
	F4 5′-TAA AAG CAT TAR TRG AAA TYT GTR CAK GTC	13%
	F5 5′-AAG CAT TAR TRG AAA TYT GTR CAK GGC	10%
	Rev 5′-CCT AAT TGA ACT TCC CAG AAG TCT TG	-
	5′-*FAM*-TTG GGC CTG AAA A“T”C CAT ACA ATA CTC CAG TAT TT	-
K65R	F1 5′- ACA ATA CTC CAR TAT TTG CCA TAA RCA G	-
	Rev 5′-CCT GGT GTC TCA TTG TTT ATA CTA GGT	-
	P1 5′-*FAM*- TCA GAG AAC “T” TAA TAA RAG AAC TCA AGA CTT CTG GGA	80%
	P2 5′-*FAM*-TCA GAG AAC “T” CAA TAA GAG AAC TCA AGA CTT CTG GGA	20%
K70R	Rev1^a^ 5′- GTT CTC TRA AAT CTA YTA WTT TTC TCC CTC	70%
	Rev2 a 5′-TTC TCT RAA ATC TAY TAW TTT TCT CCC CC	30%
	Fwd 5′- AGA RAT TTG TAC AGA RAT GGA AAA GGA AG	-
	5′-*FAM*-TTG GGC CTG AAA A“T”C CAT ACA ATA CTC CAG TAT TT	-
K103N	F1 5′-TCC HGC AGG GTT AAA RAA GGA C	40%
	F2 5′-ACA TCC MGC AGG GTT AAA AMA GGA T	27%
	F3 5′-CAT CCM GCA GGG TTA AAR VAG GAT	11%
	F4 5′-CAT CCI GCA GGI TTA AAA AAG GGC	10%
	F5 5′- T CCC KCW GGG TTA ARA AGG GAC	12%
	Rev 5′-TGG TGT CTC ATT GTT TRT ACT AGG TA	-
	5′- com.3P 5′-FAM-TGG ATG TGG GTG A“T”G CAT ATT TTT CAR TTC CCT TA	
Y181C	F1 5′-AGR AAA CAA AAY CCA GAM ATA RTT GGC TG	35%
	F2 5′- ARA AAA CAA AAY CCA GAM ATA RTT GGA TG	20%
	F3 5′-AGR AAA CAA AAY CCA GAT MTA RTT GGC TG	15%
	F4 5′- ARA AAA AAA AAY CCA GAC MTA RTT GGC TG	10%
	F5 5′-AAA ACA AAA YCC AGA RAT ART CGG CTG	10%
	F6 5′-AAA ACA AAA YCC AGA RAT ART SGG CTG	10%
	Rev 5′-ATC AGG ATG GAG TTC ATA ACC CA	-
	P1 5′-*FAM*-TAG GAT CTG ACT TAG AAA “T” AGG RCA GCA TAG ARC	80%
	P2 5′-*FAM*-TAG GAT CTG ATT “T” AGA AAT AGG RCA GCA TAG ARC	20%
M184V	F1 5′-AAA TCC ARA MMT ART TAT MTR TCA GCA CG (ID No. 33)	55%
	F2 5′-AAA TCC ARA MAT AGW RAT MTR TCA GCA CG (NEW)	25%
	F3 5′-AAA YCC ARA MAT ART TAT CTR YCA GCA TG (ID No. 35)	20%
	Rev 5′- ATC AGG ATG GAG TTC ATA ACC CA	
	P1 5′-*FAM*-TAG GAT CTG ACT TAG AAA “T” AGG RCA GCA TAG ARC	
	P2 5′-*FAM*-TAG GAT CTG ATT “T” AGA AAT AGG RCA GCA TAG ARC	
T215Y*	Rev1 a 5′-CTT TCT GAT GTT TYT KGT CTG GTG GAT	20%
	Rev2 a 5′-TTT CTG ATG TTT YTK GTC TGG TGC GT	33%
	Rev3 a 5′-TTT CTG ATR CTT TTY GTC TGG TGC GT	22%
	Rev4 a 5′- TTT CTG ATG TTT KTT GTC TGG GGC GT	10%
	Rev5 a 5′- TTT CTG ATG CTT TYT TTC TGG TGC GT	15%
	ComFwd 5′-CTT CTG GGA AGT TCA ATT AGG AAT ACC	-
	Com 1P 5′-*FAM*-TGG ATG TGG GTG A“T”G CAT ATT TYT CAR TTC CCT TA	60%
	Com 2P 5′-*FAM*-TAC TGG ATG “T” GGG TGA TGC ATA TTT TTC ART TCC CTT A	40%
T215F#	Rev1 a 5′-TTT CTG ATG TTT YTG KTC TGG TGC GA	50%
	Rev2 a 5′-CTT TCT GAT GTT TYT GKT CTG GTG CAA	50%
	ComFwd 5′-CTT CTG GGA AGT TCA ATT AGG AAT ACC	-
	Com 1P 5′-*FAM*-TGG ATG TGG GTG A“T”G CAT ATT TYT CAR TTC CCT TA	60%
	Com 2P 5′-*FAM*-TAC TGG ATG “T” GGG TGA TGC ATA TTT TTC ART TCC CTT A	40%

*FAM*, 5-fluoro;

“”, nucleotide position where quencher is placed;

a serves as mutation-specific primer;

* includes intermediates 215D, H, and N;

# includes intermediates 215L, I and V.

The mutation-specific primers (Table 1) were designed to preferentially anneal with the targeted mutation nucleotide(s), thus having reduced affinity for wildtype sequences. To accommodate the various polymorphisms in large populations, degenerate nucleotides were placed at complementary positions in the primers. Specificity was enhanced by creating designed mismatches at nucleotide(s) -2 to -4 positions from the primer 3′-end. Furthermore, to cover the spectrum of polymorphisms present, mixtures of multiple degenerate primers were often required. Mutation-specific primer mixtures were experimentally evaluated and the ratios that best balanced differences in primer avidities and minimized cross-interference in primer annealing were selected. Each change was re-evaluated against wildtype and mutant samples. For example, the M41L assay combined seven different mutation-specific primers for the detection of both the CTT and CTC mutant codons within the polymorphic sequences of the resistant samples.

The real-time PCR probes anneal to sequences within the total copy and mutation-specific amplicons and merely act as reporters of primer extension. The probes were 5′labeled with FAM (6-carboxyfluorescein) and internally quenched with a black-hole quencher (BHQ) placed at the positions indicated by the quotation marks (“ “) in Table 1. In the probe design, the quencher was placed between 9–18 nucleotides from the 5′ FAM-labeled base at the position providing the best quenching of background fluorescence. Fluorescent signals, reported as relative fluorescent units (RFU), were generated by degradation of the fluorescent probes which resulted in the separation of the fluorophore from the quencher during each round of chain elongation. The threshold for each test was set at an RFU level that corresponded to the beginning of the log phase of the amplification curves. For additional information on the use of probes and interpretation of ΔCT see the supporting information on assay design in Method S1.

Real-time PCRs were initiated with a hot-start incubation at 94°C for 11 minutes before proceeding to 45 cycles of melting at 94°C for 30 seconds, annealing at 50°C for 15 seconds and extension at 60°C for 30 seconds. All reactions were performed in a total volume of 50 µL/well in 96-well PCR plates using iCycler real-time PCR thermocyclers with optical units (Bio-Rad) and AmpliTaq Gold polymerase (2.5 U/reaction; Applied Biosystems). Final reagent concentrations were 320 nM for the forward and reverse primers, 160 nM probe(s), and 400 µM dNTPs. Low viral load samples that generated total copy CTs which appeared after 26 cycles sometimes yielded false-positive results. To avoid this complication, all samples with CTs above 26 cycles were further amplified by nested PCR prior to real-time PCR testing. To adequately subtract background fluorescence, high virus load samples that produced total copy CTs appearing less than 10 cycles were diluted 1∶100–1000 in RNase/DNase-free reagent-grade water and retested. We found that 1∶20 dilutions of RT-PCR products from all but the samples with virus loads below 5000 copies/ml provided adequate template for real-time PCR testing.

### Assay evaluations on plasmids and clinical specimens

Each mutation-specific primer mixture was initially evaluated against both cloned lab-generated and patient-derived mutant virus sequences that were serially diluted 10-fold in backgrounds of wildtype sequence plasmids. The supporting information on plasmid evaluations in Method S2 describes the use of cloned virus sequences for the preliminary selection of mutation-specific primers and determining assay absolute detection limits.

To both evaluate the frequency of natural polymorphisms at codons associated with drug resistance and establish assay cutoffs for screening subtype B virus infections, we tested 138 serum samples collected from 117 individuals infected with HIV-1 in the US between 1982–1985, prior to the era of antiretroviral drug use. Within these early HIV specimens were longitudinal samples from 23 individuals which were examined for evidence of polymorphic changes over time. Assay sensitivities for clinical screening were determined using samples from a total of 302 individuals with drug resistance mutations detectable by bulk sequence genotyping. The resistance mutation samples were collected in the US and Canada during 1998–2005, and included a portion of US samples from a previously reported surveillance study [19]. We evaluated archived specimens which the CDC Institutional Review Board determined did not involve research on identifiable subjects. Samples with resistance mutations were obtained from 51 individuals with protease L90M, and those with reverse transcriptase mutations included 78 subjects with M41L, 26 with K65R, 59 with K70R, 81 with K103N, 28 with Y181C, 67 with M184V, 44 with T215Y, and 35 with T215F. To increase the stringency of assay evaluations, specimens with substantial numbers of polymorphisms in primer binding sites were included.

For the purpose of clinical testing, each assay cutoff was placed at a ΔCT that excluded all the early wildtype virus samples and, when possible, was at least one amplification cycle less than the lowest pre-antiretroviral wildtype ΔCT as an added buffer against non-specific reactivity. It is expected that the sensitivities observed with plasmid dilutions may not be achievable for some clinical samples because of low virus copy numbers or genomic differences which affect primer performance.

### Low-frequency mutation detection

After validating the assays on early wildtype and known mutant viruses, we sought to demonstrate the ease with which the sensitive assays could detect low-frequency mutations. Tests for five resistance mutations were applied to a small assortment of convenient mutant virus clinical samples previously used in assay validation that had no evidence of the targeted mutation by standard genotyping. Tests for L90M, K103N, Y181C, M184V and T215Y were applied to 30, 19, 13, 11 and 10 specimens, respectively.

### Assessing mutation associations in mutation-specific amplicons

To overcome the single mutation detection limitation of point-mutation assays, we evaluated whether additional information on resistance mutations could be gained from the real-time PCR assays. To address this, we performed direct sequencing (BigDye reagent, Prism 3130XL analyzer, Applied Biosystems) of the products from positive mutation-specific reactions and compared these to their respective sample bulk sequence for evidence of nucleotide differences. In order to include other codons of interest with the K103N test, we extended the amplicon by using the 184V REV reverse primer (Table 1), which allowed sequencing to RT codon 221. Any other resistance mutation(s) found in the mutation-specific amplicon would indicate that they were on the same viral strand(s) as the mutation that was specifically targeted in the reaction. The absence of nucleotide mixtures at resistance codons within mutation-specific amplicons is informative because it indicates the targeted mutation is entirely associated with those mutations. Conversely, nucleotide mixtures suggest that the interrogated mutation is linked to more than one resistance genotype.

### Clonal sequencing

To verify mutation associations depicted in the sequences of mutation-specific amplicons, nested amplifications of the original RT-PCRs were cloned into the TA vector (Invitrogen) for *E. coli* transformation [3]. Colonies were screened with the same assay used for the low frequency mutation detection and positive clones were sequence analyzed as above.

## Results

### Assay sensitivities on cloned virus sequences

**Figure 2:**
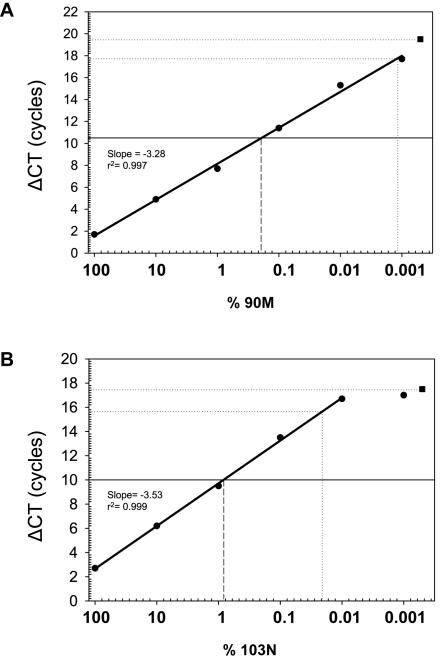
Mutation-specific assay reactivity on plasmids. Cloned L90M (A.) and K103N (B.) mutant virus sequence was diluted 10-fold, from 100% to 0.001%, in backgrounds of wildtype sequence to determine assay detection limits. Plotted are the mean ΔCT versus log_10_ of the mutant dilution series (•), and the mean ΔCT for wildtype sequence alone (▪). The lower detection limit (lower dotted line) was placed at the ΔCT equivalent to 0.5 log_10_ below (0.5-log greater reactivity than) the wildtype ΔCT. Dilutions that fall outside the linear range are not considered. For comparison, the mutant virus frequency equivalences for the established clinical cutoffs are also shown (dashed line).

Relative limits of detection were compared in a simple laboratory setting using serial dilutions of cloned mutant template. The ΔCT that was equivalent to a 0.5 log greater reactivity than the wildtype mean ΔCT on the dilution curve was used to compare assay sensitivities (Figure 2). This approach yielded detection limits of 0.001% and 0.02% for L90M and K103N, respectively. Absolute detection limits for the remaining assays were likewise determined on cloned control samples and the corresponding frequencies found to be 0.02% for M41L, 0.05% for K70R, 0.08% for K65R, Y181C, M184V, and T215F, and 0.2% for T215Y (Table 2).

**Table 2:** Assay ΔCT measures, cutoffs, and sensitivities on clinical samples.

Assay	ΔCT cutoff (# cycles)	Cutoff mean % mutant equivalence	Sensitivity, #Pos/mutants tested (%)	Mean ΔCT (range) of pre-ART wildtype n = 138	Mean ΔCT (range) of mutant samples	False-negatives, ΔCTs
L90M	10.5	0.4	51/51 (100)	16.8 (12.0–28.0)	0.9 (−9.1–5.2)	-
M41L	10.0	0.8	76/78 (97)	16.4 (11.2–21.0)	4.4 (−5.8–10.0)	12.1, 16.5
K65R	8.5	0.3	26/26 (100)	10.9 (9.1–11.8)	1.3 (−0.4–5.8)	-
K70R	7.0	2.0	57/59 (97)	11.6 (7.2–20.1)	2.2 (−2.6–6.2)	7.4, 9.0
K103N	10.0	0.9	80/81 (99)	15.7 (10.2–25.0)	5.8 (2.7–9.7)	11.3
Y181C	10.0	1.0	27/28 (96)	14.3 (11.2–21.1)	6.4 (3.1–9.6)	12.6
M184V	8.5	0.5	65/67 (97)	11.6 (8.7–30.9)	5.0 (1.2–8.2)	9.8, 11.9
T215Y*	10.5	1.0	44/44 (100)	13.9 (11.5–16.4)	6.0 (2.4–9.6)	-
T215F#	10.5	0.7	35/35 (100)	14.4 (11.9–23.8)	3.6 (1.2–5.8)	-

Pre-ART, pre-antiretroviral drug use;

* includes intermediates 215D, H and N;

# includes intermediates 215L, I, and V.

### Assay sensitivity and performance with clinical samples

The viral RNA extraction from plasma followed by the RTP-F1- RTP-REV2 RT-PCR could amplify as little as 10 input RNA copies when using the total copy primers to detect amplified product (data not shown). In some cases, as little as 5 RNA copies could be amplified with the RTP-F2 and RTP-REV2 RT-only primer pair. However, to obtain sufficient amplification with both the total copy and mutation-specific reactions with majority mutant virus samples, around 20–80 input RNA copies were required depending on the assay.

Assay cutoff values intended for population-wide clinical screening were established using 138 patient-derived wildtype specimens collected before the era of ARV drug treatment. Following the selection of each assay cut-off, assay sensitivity was evaluated using a total of 302 samples with sequence-detectable drug resistance mutations.

**Figure 3:**
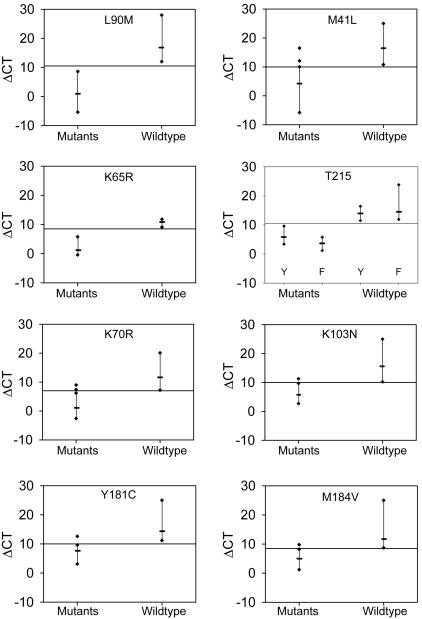
Assay reactivities with clinical samples having sequence-detectable mutations and with pre-antiretroviral wildtype virus samples. The range of reactivity for each assay is shown for wildtype and mutant samples. The upper and lower ΔCT and the mean (hash) for each group are indicated. Assay cutoffs (horizontal line) were established to exclude all wildtype viruses from the pre-antiretroviral era.

With some longitudinal wildtype samples collected in the pre-ARV drug era, we observed ΔCTs that differed as much as 6.5 cycles between time points. The greatest ΔCT decrease was seen with K70R, which resulted in this assay having the narrowest window of mutation detection (Figure 3). The ΔCT variation with longitudinal samples suggested that mutations at positions associated with drug resistance can naturally arise and fluctuate over time within individuals. Using the L90M assay as an example, the resulting distribution of collated ΔCTs from the pre-ARV era wildtype samples supported a ΔCT cutoff of 10.5 cycles for L90M clinical testing (ΔCTs ranged from 12.0–28.0 cycles) (Figure 3). Extrapolating from the dilution curve for cloned L90M sequences, this placement corresponded to a frequency mean of 0.2% mutant virus (see Figure 2). At this cut-off, all 51 genotyped 90M samples were positive (ΔCTs ranged from -9.1 to 5.2 cycles) (Table 2). The tests for mutations in the HIV-1 reverse transcriptase were likewise evaluated and the resulting assay limits and performances are provided in Table 2 and Figure 3. For each assay, the mean ΔCTs of the specimens documented to have mutations were significantly lower than the ΔCTs of the wildtype samples (all p values <0.0001, T-test).

Because of unusual polymorphisms, some samples comprised almost entirely of mutant virus produced ΔCTs near or above the cutoff. In these situations, elevated ΔCTs resulting from weak primer binding could be interpreted as mutant viruses present at low frequencies. Hence, this testing format is best-suited to provide highly specific population-level resistance screening and is not necessarily applicable to mutant virus quantitation.

### Identification of low-frequency mutations and confirmation by cloning

To demonstrate the ability of real-time PCR assays to detect drug-resistant viruses present as minor variants in specimens, assays for L90M, K103N, Y181C, M184V and T215Y were applied to assortment of clinical samples that had major resistance mutations, but had no evidence of the targeted mutation by bulk sequencing. Each assay identified at least one mutant sample with a hidden low-frequency mutation in the few samples tested. L90M was identified in 2/30 samples (ΔCTs = 1.0, 5.0 cycles), K103N in 3/19 (ΔCTs = 7.6, 7.7, 9.8), Y181C in 1/13 (ΔCT = 7.6), M184V in 2/11 (ΔCTs = 5.9, 7.3), and T215Y in 1 of 10 samples (ΔCT = 5.6). One representative low-frequency variant for each of the five mutations tested was verified by clonal sequencing which found the mutation frequencies to be between 0.7%–11%.

### Linking high and low-frequency resistance mutations

**Figure 4:**
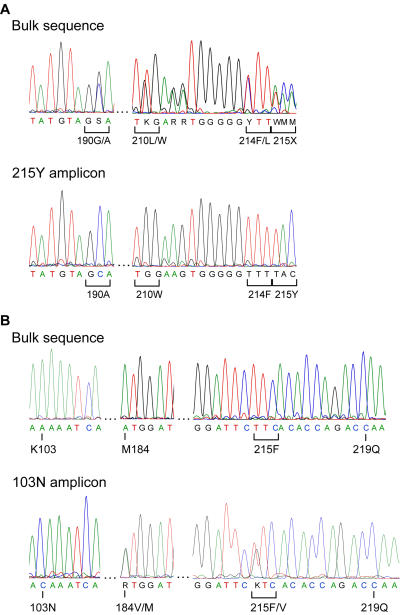
Detection of other associated resistance mutations in mutation-specific amplicons. A. The undecipherable codon 215 in the bulk sequence of this sample was resolved (positive) with the T215Y test. The sequence of the T215Y-positive amplicon showed that the mutations present in the bulk sequence were linked. B. The low-frequency K103N amplicon sequence from this sample uncovered another previously undetected mutation, M184V. 215X, undecipherable codon 215.

To overcome the point-mutation testing limitation of single mutation detection, we directly sequenced positive mutation-specific PCR products to ascertain whether additional genotypic information could be easily obtained. Two samples that demonstrate the usefulness of this approach are described. Bulk sequencing of one sample showed nucleotide mixtures in the RT at the resistance-associated codons G190G/A, L210L/W and an undecipherable mixture at codon 215 in reverse transcriptase (sample ‘A’) (Figure 4A). This sample was tested with both the T215Y and T215F PCR assays, but was found to be positive for only the T215Y mutation (ΔCT = 4.1). Direct sequencing of the T215Y-positive amplicon revealed that both the G190A and L210W mutations were present as unmixed codons (Figure 4A). The mutation-specific sequence resolved the resistance mutation at codon 215 and suggested all three mutations (G190A, L210W, and T215Y) were linked on the same genome. The co-linkage of all three mutations was confirmed in 6 of 6 T215Y-positive clones. A second sample (sample ‘B’, Figure 4B) that had only T215F and K219Q mutations by bulk genotyping was positive for low-frequency K103N using the extended K103N assay (ΔCT = 9.8). The sequence of the K103N-positive amplicon contained the K219Q mutation and a T215F/V mixture. Also in this amplicon was another low-frequency mutation, M184V, which appeared as a 50% mixture with wildtype sequence. Hence, the K103N-specific sequence showed that low-frequency K103N mutants were associated with K219Q, M184V and M, and T215F and V. Clonal sequencing confirmed K103N in 3/81 clones (4%) obtained from the sample and all 3 clones verified K103N was linked with K219Q. Two of the clones also had the M184V and T215V mutations. The two clonal genotypes are provided in Figure S1. The ability to resolve codon 219 in the extended K103N mutation-specific sequence demonstrated that resistance mutations as far apart as 117 amino acids could be evaluated for linkage.

## Discussion

We describe a simple and sensitive approach for mutant virus screening that is able to detect drug-selected resistance mutations at frequencies as low as 0.3% in clinical samples, allowing for the identification of minority HIV-1 variants. The real-time PCR-based point mutation assays were robust with the 474 total subtype B virus specimens evaluated, supporting their use for clinical testing. Improved low-frequency mutation detection was provided by clinical testing cutoffs that were 10–67-fold more sensitive than conventional sequencing. These cutoffs were above the background reactivities observed with drug-naïve wildtype HIV collected in the pre-antiretroviral drug era and, thus, identify mutations occurring at frequencies above those found naturally in virus quasispecies. Although this paper focused on resistance mutation testing in subtype B viruses, we earlier demonstrated that real-time PCR assays can also be successfully developed for subtype C viruses which are globally the most prevalent [3]. When possible, oligonucleotides are designed so that they might also be used with more than one subtype; however, screening for resistance in non-B subtypes requires that the oligonucleotides are properly validated for those subtypes.

Setting stringent assay cutoffs to avoid detecting natural polymorphisms resulted in primer designs that provided sensitivities of 96% to >99% and specificities of >99% with samples that included highly polymorphic sequences (Table 2). Although the real-time PCR assays were able to detect as little as 0.001%–0.2% cloned mutant sequences, increasing ΔCT cutoffs to expand the mutation detection range would make it difficult to differentiate drug-selected mutants from naturally-occurring variants. However, in antiretroviral studies of infected persons in which pre-drug exposure samples are available, a comparative method could be used to evaluate drug resistance rather than an absolute ΔCT cut-off. In these settings, a substantial decrease in ΔCT between the pre- and post-treatment samples for an individual could indicate the emergence of a mutation even if the ΔCT does not drop below the cut-off established for screening. Furthermore, in experimental settings where baseline genotypes are known, individual primers that best match the virus sequence may be used, instead of mixtures, to maximize assay sensitivity.

Evidence of improved resistance mutation detection was found in testing only a few samples which uncovered hidden mutations. However, to overcome the limitation of single mutation detection, we directly sequenced mutation-specific reactions as a simple way to rapidly assess mutation associations and demonstrated that the genotypic findings were similar to that obtained by cloning virus templates. Sequencing mutation-specific amplicons also identified additional low-frequency drug resistance mutations when they were linked to the targeted mutation, as was seen with the discovery of M184V in sample B. Therefore, previously hidden multi-drug resistance could easily be uncovered.

Sensitive testing can be streamlined by using a tailored and concise panel of mutation-specific tests that span the protease and RT regions, followed by sequencing the mutation-specific amplicons from positive tests to evaluate for linked mutations. This would allow for sensitive primary screening of resistance as well as the identification of other mutations present in the individual. The capacity to identify linked mutations could be important for understanding the persistence [20] and clinical impact of mutant variants.

In conclusion, we present a panel of real-time PCR assays that provide a sensitive and user-friendly method for screening HIV-1 drug resistance mutations. The substantial oligonucleotide modifications that allowed for successful detection of mutations within diverse sequence backgrounds, combined with extensive validation and improved sensitivity, make these assays feasible for large-scale resistance testing. Furthermore, coupling mutation-specific sequencing to sensitive screening expands the capability of point-mutation testing and provides a powerful approach for studying the dynamics and clinical consequences of drug-resistant HIV-1. The simplicity of this methodology and the abundance of real-time PCR materials currently make sensitive PCR assays more practical for broader drug resistance testing than the more complex and expensive testing methods.

## Supporting Information

Figure S1 The HXB2 RT nucleotides, bulk sequence of sample B, and sample B low-frequency K103N clones are shown. The sites of the codon 103, 184, 215 and 219 resistance-associated nucleotides are underlined (_). Dots (.) over the sequences indicate nucleotides that differ from HXB2. A ‘C’ at 103 = K103N, a ‘G’ at 184 = M184V, a ‘TT’ at 215 = T215F, a ‘GT’ at 215 = T215V, and a ‘C’ at 219 = K219Q.
(0.01 MB PDF)

Method S1 PCR assay design.
(0.03 MB DOC)

Method S2 Assay evaluations on plasmids.
(0.03 MB DOC)
