# Supplementary material for: Simple PCR Assays Improve the Sensitivity of HIV-1 Subtype B Drug Resistance Testing and Allow Linking of Resistance Mutations
Source: PLoS One. 2007 Jul 25;2(7):e638. doi: 10.1371/journal.pone.0000638 (PMC1919426; doi:10.1371/journal.pone.0000638)
Supplement: Figure S1 — The HXB2 RT nucleotides, bulk sequence of sample B, and sample B low-frequency K103N clones are shown. The sites of the codon 103, 184, 215 and 219 resistance-associated nucleotides are underlined (_). Dots (.) over the sequences indicate nucleotides that differ from HXB2. A ‘C’ at 103 = K103N, a ‘G’ at 184 = M184V, a ‘TT’ at 215 = T215F, a ‘GT’ at 215 = T215V, and a ‘C’ at 219 = K219Q. (0.01 MB PDF) [file pone.0000638.s001.pdf]

```
                  .                 .        .          .    .           .
HXB2 RT   (307) AAAAAATCAGTAACAGTACTGGATGTGGGTGATGCATATTTTTCAGTTCCCTTAGATGAA
BULKSEQ         AAAAAATCAGTAACAGTACTAGATGTGGGKGATGCATATTTTTCAGTTCCCTTAGATAAA
clone 1         AACAAATCAGTAACAGTACTAGATGTGGGTGATGCATATTATTCAATTCCCTTAGATAAA
clone 2         AACAAATCAGTAACAGTACTAGATGTGGGTGATGCATATTATTCAGTTCCCTTAGATGAA
                103
                  .                                 .
HXB2 RT   (367) GACTTCAGGAAGTATACTGCATTTACCATACCTAGTATAAACAATGAGACACCAGGGATT
BULKSEQ         GAMTTCAGGAAGTATACTGCATTTACCATACCTAGTGTAAACAATGAGACACCAGGGATT
clone 1         GACTTCAGGAAGTATACTGCATTTACCATACCTAGTGTAAACAATGAGACACCAGGGATT
clone 2         GACTTCAGGAAGTATACTGCATTTACCATACCTAGTGTAAACAATGAGACACCAGGGATT

                    .  ...
HXB2 RT   (427) AGATATCAGTACAATGTGCTTCCACAGGGATGGAAAGGATCACCAGCAATATTCCAAAGT
BULKSEQ         AGATATCAGTACAATGTGCTGCCMCAGGGATGGAAAGGATCACCAGCAATATTCCAAAGT
clone 1         AGATATCAGTACAATGTGCTGCCACAGGGATGGAAAGGATCACCAGCAATATTCCAAAGT
clone 2         AGATATCAGTACAATGTGCTGCCCATGGGATGGAAAGGATCACCAGCAATATTCCAAAGT

                            .               .
HXB2 RT   (487) AGCATGACAAAAATCTTAGAGCCTTTTAGAAAACAAAATCCAGACATAGTTATCTATCAA
BULKSEQ         AGCATGACAAAAATCTTAGAGCCTTTTARAAAACAAAATCCAGACATAGTTATCTATCAA
clone 1         AGCATGACAAAAATCTTAGAGCCTTTTAGAAAACAAAATCCAGACATAGTTATCTATCAA
clone 2         AGCATGACAAAAGTCTTAGAGCCTTTTAGAAAACAAAATCCAGACATAGTTATCTATCAA

                   .       .                            .          .
HXB2 RT   (547) TACATGGATGATTTGTATGTAGGATCTGACTTAGAAATAGGGCAGCATAGAACAAAAATA
BULKSEQ         TACATGGATGACTTGTATGTAGGATCTGACTTAGAAATAGAGCAGCATAGAGCAAAAATA
clone 1         TACATGGATGACTTGTATGTAGGATCTGACTTAGAAATAGAGCAGCATAGAGCAAAAATA
clone 2         TATGTGGATGACTTGTATGTAGGATCTGACTTAGAAATAGAGCAGCATAGAGCAAAAATA
                   184
                .    .      . ..          .      . ...          .
HXB2 RT   (607) GAGGAGCTGAGACAACATCTGTTGAGGTGGGGACTTACCACACCAGACAAA
BULKSEQ         AAGGAACTGAGAGACTATCTGTTGAGATGGGGATTCTTCACACCAGACCAA
clone 1         AAGGAACTGAGAGACTATCTGTTGAGATGGGGATTCTTCACACCAGACCAA
clone 2         AAGGAACTGAGAGACTATCTGTTGAGATGGGGATTCGTCACACCAGACCAA
                                                    215         219
```
