# Supplementary material for: Simple PCR Assays Improve the Sensitivity of HIV-1 Subtype B Drug Resistance Testing and Allow Linking of Resistance Mutations
Source: PLoS One. 2007 Jul 25;2(7):e638. doi: 10.1371/journal.pone.0000638 (PMC1919426; doi:10.1371/journal.pone.0000638)
Supplement: Method S1 — PCR assay design. (0.03 MB DOC) [file pone.0000638.s002.doc]

**Method S1.**

**PCR assay design**

We found that a fluorescent probe coupled to a black hole quencher (BHQ) as a reporter of PCR amplification often allowed for better discrimination between wildtype and mutant virus sequences than did a DNA intercalating dye (e.g., SybrGreen, Applied Biosystems) (data not shown). The use of longer probes to strengthen annealing was possible when placing the quencher at an internal nucleotide residue for optimal quenching of the FAM (6-carboxyfluorescein) fluorophore. Furthermore, the use of probe mixtures also helped to overcome sequence polymorphisms present at annealing sites.

The various mutation-specific primer sets differed slightly in their affinities for mutant sequences relative to the total copy reactions. This sometimes caused mutation-specific CTs to appear earlier than the total copy CT (generating negative ΔCT values); however, this did not impact our ability to define the mutation-positive range for the assays or interpret resistance mutations in samples.
