# Supplementary material for: Simple PCR Assays Improve the Sensitivity of HIV-1 Subtype B Drug Resistance Testing and Allow Linking of Resistance Mutations
Source: PLoS One. 2007 Jul 25;2(7):e638. doi: 10.1371/journal.pone.0000638 (PMC1919426; doi:10.1371/journal.pone.0000638)
Supplement: Method S2 — Assay evaluations on plasmids. (0.03 MB DOC) [file pone.0000638.s003.doc]

**Method S2.**

**Assay evaluation on plasmids**

Mutant and wildtype plasmids were used in the preliminary selection of primer mixtures that provided the greatest sensitivity and specificity. The absolute mutation detection limits for the primer mixtures, that is, the greatest ΔCT that was able to distinguish mutant viruses from wildtype, were estimated from triplicate testing of the mutant clone dilutions. The assays evaluated mutation-containing sequences at frequencies between 100%-0.001%, with each dilution having a total of 10^6^ plasmid copies. The ΔCTs generated from the mutant dilutions were compared to the ΔCTs generated with the wildtype plasmids alone. Solely for the purpose of comparing relative assay detection limits with finite virus sequences, a ΔCT within the linear dilution range that was equivalent to a frequency increase of 0.5 log_10_ above the wildtype mean ΔCT was chosen as the absolute assay detection limit. Selecting the detection limit in this manner provided an adequate buffer against variability in wildtype sequences and also took into account the PCR efficiency of the assay (slope of the dilution curve).
